# Patient-Specific Modelling and Parameter Optimisation to Simulate Dilated Cardiomyopathy in Children

**DOI:** 10.1007/s13239-022-00611-9

**Published:** 2022-02-22

**Authors:** Selim Bozkurt, Waleed Paracha, Kaushiki Bakaya, Silvia Schievano

**Affiliations:** 1 School of Engineering, Ulster University, Newtownabbey, UK; 2 Institute of Cardiovascular Science, University College London, London, UK; 3 University College London Medical School, London, UK

**Keywords:** Lumped parameter model, Patient-specific cardiac function, Dilated cardiomyopathy, Paediatrics

## Abstract

**Purpose:**

Lumped parameter modelling has been widely used to simulate cardiac function and physiological scenarios in cardiovascular research. Whereas several patient-specific lumped parameter models have been reported for adults, there is a limited number of studies aiming to simulate cardiac function in children. The aim of this study is to simulate patient-specific cardiovascular dynamics in children diagnosed with dilated cardiomyopathy, using a lumped parameter model.

**Methods:**

Patient data including age, gender, heart rate, left and right ventricular end-systolic and end-diastolic volumes, cardiac output, systolic and diastolic aortic pressures were collected from 3 patients at Great Ormond Street Hospital for Children, London, UK. Ventricular geometrical data were additionally retrieved from cardiovascular magnetic resonance images. 23 parameters in the lumped parameter model were optimised to simulate systolic and diastolic pressures, end-systolic and end-diastolic volumes, cardiac output and left and right ventricular diameters in the patients using a direct search optimisation method.

**Results:**

Difference between the haemodynamic parameters in the optimised cardiovascular system models and clinical data was less than 10%.

**Conclusion:**

The simulation results show the potential of patient-specific lumped parameter modelling to simulate clinical cases. Modelling patient specific cardiac function and blood flow in the paediatric patients would allow us to evaluate a variety of physiological scenarios and treatment options.

## Introduction

Lumped parameter modelling has been widely adopted to simulate cardiac function and physiological scenarios in cardiovascular research as they allow to study complex conditions in a simplified manner. For instance, heart failure has been simulated using this method by reducing systolic elastance, which describe the relationship between left ventricular pressure and volume.^2^ Ventricular wall mechanics have been modelled using active and passive stresses on a fibre which in turn have been utilised to simulate and heart failure and mechanical circulatory support in adults.^5^ Multi-scale models which include cellular, protein and organ level dynamics have also been utilised to simulate heart failure in adults.^1^ Atrial function in adults have been simulated using a multi-scale model.^25^ Dynamics of heart valves have been modelled to simulate blood flow through the healthy and diseased heart valves in adults.^16^ Lumped parameter models simulating heart failure or heart valve dynamics have been utilised to evaluate treatment techniques such heart pump support in adults. For instance, Gross et al.^12^ evaluated the effect of left ventricular assist device speed increase during exercise in a model simulating heart failure. Liu et al.^20^ evaluated varying speed modulation algorithms for continuous-flow left ventricular assist devices based on a cardiovascular coupling numerical model. Modelling of cardiac function and simulation of physiological cases in adults have been studied extensively and detailed information can be found in the literature.^18^

Lumped parameter models simulating patient-specific cardiac dynamics in adults have already been developed. For instance, Ellwein et al.^7^ developed a patient-specific model of cardiovascular and respiratory systems during hypercapnia. Pope et al.^26^ showed how sensitivity analysis and subset selection can be employed in a cardiovascular model to estimate system parameters for healthy young and elderly subjects. Neal and Bassingthwaighte^22^ developed a method to estimate subject-specific cardiac output and blood volume during haemorrhage. There is a limited number of studies aiming to simulate physiological cases in children where there is a need for such models. Schiavazzi et al.^29^ estimated parameters of patient-specific in single-ventricle lumped circulation models under uncertainty. Goodwin et al.^10^ developed a model simulating infant cardiovascular physiology for educational purposes. Sá Couto et al.^28^ developed a cardiovascular system model to simulate neonatal physiology. Giridharan et al.^9^ developed a computer model of the paediatric circulatory system to test ventricular assist devices. Petukhov and Telyshev^24^ developed a cardiovascular system model for paediatric patients with congenital heart diseases. Bozkurt^3^ developed a cardiovascular system model capable of simulating cardiac function and heart chamber dimensions over a cardiac cycle in adults and children.

Mostly, average models describing generalised physiology in adults and children have been utilised to simulate physiological scenarios such as heart failure. However, causes of heart failure vary significantly from patient to patient and indeed, several different therapies, including digoxin, beta-blockers, diuretics or angiotensin-converting enzyme inhibitors, are used to improve heart failure.^14^ Moreover, in children, clinical decisions are often dependant on results recorded in adult heart failure trials.^6^ Therefore, treatment is negatively affected as there are substantial difficulties in adapting adult data to paediatric patients. Patient-specific numerical models simulating cardiac function may help to evaluate treatment options for children. Our previous model^3^ presents a concept model in which the parameters were not optimized to simulate patient-specific clinical data. It simulates hemodynamic variables in adults and children within a physiological interval. The aim in this study is to simulate patient-specific cardiovascular dynamics in children diagnosed with dilated cardiomyopathy using a new optimisation strategy which can be applied without computational cost for parameter identification.

## Materials and Methods

The following demographics and hemodynamic parameters were collected from 3 patients diagnosed with dilated cardiomyopathy at Great Ormond Street Hospital for Children, London, UK, by searching the clinical database: age, gender, heart rate, cardiac output, left and right ventricular end-systolic and end-diastolic volumes, systolic and diastolic aortic pressures. The patients were selected to simulate mild and severe dilated cardiomyopathy in the left ventricle and biventricular dysfunction.

Cardiac magnetic resonance images (1.5-Tesla Magnetom Avanto scanner, Siemens Medical Solutions, Erlangen, Germany) were reviewed for these patients. The steady-state free precession sequences acquired at both end-diastole, and end-systole, were used to measure cardiac dimensions in Simpleware ScanIP 2018 (Synopsis, CA, USA). Four-chamber view was used to measure left and right ventricular long axis length and right ventricular basal diameter; the short-axis view was used to measure left ventricular lateral-septal diameter.

### Patient Information

In children, dilated cardiomyopathy is diagnosed considering z-scores for ventricular ejection fraction, ventricular volume and ventricular end-diastolic and end-systolic diameters indexed on body surface area and measurements of the ventricular systolic function (z-score>2).^19,31^

Patient 1 (male, age at scan = 8 years 7 months, body surface area = 0.94 m^2^) was diagnosed with mild dilated cardiomyopathy. There were no signs of left ventricular hypertrophy, atrial dilation, atrio-ventricular valve regurgitation, and outflow tracts’ obstruction. Both aortic and pulmonary valves were competent, with no stenosis. The right ventricle had normal global systolic function and volume. Cardiac output was 5.3 L/min, and systolic and diastolic pressures were 109 mmHg and 60 mmHg, respectively. End-diastolic volume was 106 mmHg (z-score=2.1).

Patient 2 (female, age at scan = 10 years 8 months, body surface area = 0.94 m^2^) was diagnosed with severe dilated cardiomyopathy. She presented with global thinning of the myocardium with no left ventricular hypertrophy. There was no left atrial dilatation and no mitral regurgitation on volume measurements. There was no left ventricular outflow tract obstruction and the aortic valve had normal function. The right atrium was not dilated. There was no tricuspid valve regurgitation. There was no right ventricular outflow tract obstruction. The pulmonary valve was competent with no stenosis. Cardiac output was 2.6 L/min, and systolic and diastolic pressures were 84 mmHg and 49 mmHg, respectively.

Patient 3 (male, age at scan = 15 years 2 months, body surface area = 1.44 m^2^) was diagnosed with severe cardiomyopathy with biventricular dysfunction and raised pulmonary pressure. The right atrium was dilated, whilst the left atrial chamber remained normal. There was 37% tricuspid valve regurgitation. The right ventricular myocardium was moderately hypertrophied, with globally poor contractility and evidence of severely impaired diastolic function. The right ventricle was dilated at both end-diastole and end-systole. There was no left ventricular hypertrophy. The left ventricular chamber was of low-normal body surface area indexed volume at end-diastole, and normal volume at end-systole, appearing under-filled, with a global impairment of systolic function. The pulmonary valve was unobstructed whilst there was 11% pulmonary valve regurgitation. The aortic valve was competent. Cardiac output was 2.6 L/min, and systolic and diastolic pressures were 118 mmHg and 64 mmHg, respectively.

### Cardiovascular System Model

The cardiovascular system model used in this study simulates pressures, volumes and diameters in the heart chambers, flow rate through the heart valves, and pressures and flow rates in the systemic and pulmonary circulations. The left ventricular pressure (*p*_*lv*_) was described using the active and passive contraction components (*p*_*lv,a*_, *p*_*lv,p*_). The ventricular active pressure component was described using systolic ventricular elastance (*E*_*es,lv*_), ventricular volume and zero-pressure volume (*V*_*lv*_, *V*_*lv,0*_), and the activation function (*f*_*act,lv*_). The ventricular passive pressure component (*p*_*lv,p*_) was modelled using an exponential relationship including volume (*V*_*lv*_) and additional coefficients (*A*, *B*). Detailed information about the cardiovascular system model can be found in.^3^

1 $$p_{lv} = p_{lv,a} + p_{lv,p}$$

2 $$p_{lv,a} = E_{es,lv} (V_{lv} - V_{lv,0} )f_{act,lv} (t)$$

3 $$p_{lv,p} = A\left[ {e^{{(BV_{lv} )}} - 1} \right]$$

Left ventricular volume (*V*_*lv*_) was described using the left ventricular radius (*r*_*lv*_), long axis length (*l*_*lv*_) and an additional coefficient (*K*_*lv*_), which includes effects of the contraction in the long axis and scales the proportion between the left ventricular radius and volume over a cardiac cycle. Change of the left ventricular radius (*r*_*lv*_) were described utilising the flow rates through the aortic and mitral valves (*Q*_*av*_, *Q*_*mv*_), the left ventricular volume (*V*_*lv*_*)* and long axis length (*l*_*lv*_), and the coefficient *K*_*lv*_.

4 $$V_{lv} = \frac{2}{3}\pi K_{lv} r_{lv}^{2} l_{lv}$$

5 $$\frac{{dr_{lv} }}{dt} = \frac{{3(Q_{mv} - Q_{av} )}}{{4\pi K_{lv} l_{lv} }}\left( {\frac{{3V_{lv} }}{{2\pi K_{lv} l_{lv} }}} \right)^{ - 1/2}$$

The left atrial pressure (*p*_*la*_) and volume (*V*_*la*_) relationship was described using the elastance (*E*_*la*_).

6 $$p_{la} = E_{la} (t)(V_{la} - V_{la,0} )$$

The left atrial volume (*V*_*la*_) was described using the left atrial radius (*r*_*la*_) and long axis length (*l*_*la*_), and an additional coefficient (*K*_*la*_). Changes of the left atrial radius (*r*_*la*_) were described utilising the flow rates through the mitral valve and pulmonary vein (*Q*_*mv*_, *Q*_*vp*_), the left atrial volume (*V*_*la*_) and long axis length (*l*_*la*_), and the coefficient *K*_*la*_.

7 $$V_{la} = \frac{2}{3}\pi K_{la} r_{la}^{2} l_{la}$$

8 $$\frac{{dr_{la} }}{dt} = \frac{{3(Q_{vp} - Q_{mv} )}}{{4\pi K_{la} l_{la} }}\left( {\frac{{3V_{la} }}{{2\pi K_{la} l_{la} }}} \right)^{ - 1/2}$$

The right atrium and ventricle were modelled in the same way as the left atrium and ventricle, using the different parameter values.

Heart valves were modelled using the pressure across the valve and the characteristic resistances for the forward and backward flows (*R*_*mv,f*_, *R*_*mv,b*_) to simulate regurgitant valve flow in the patients. The flow rate expression through the mitral valve is provided below.

9 $$Q_{mv} = \left\{ \begin{gathered} \frac{{p_{la} - p_{lv} }}{{R_{mv,f} }}\,\,\,\,\,\,\,\,\,\,\,\,p_{la} > p_{lv} \hfill \\ \frac{{p_{la} - p_{lv} }}{{R_{mv,b} }}\,\,\,\,\,\,\,\,\,\,\,\,p_{la} < p_{lv} \hfill \\ \end{gathered} \right.$$

The other heart valves were modelled in a similar way using the appropriate parameter values.

**Figure 1:**
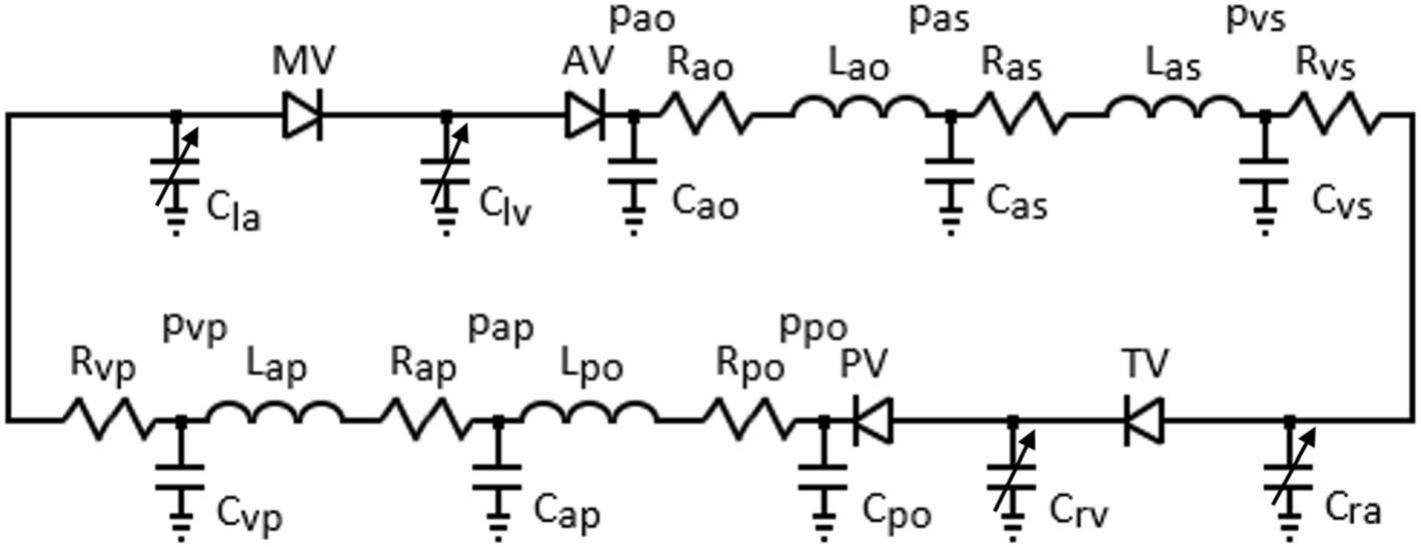
The electric analogue model of the of the cardiovascular system. *R*, *L* and *C* denote resistance, inertance and compliance, *p* and denote pressure, *MV*, *AV*, *TV* and *PV* are mitral, aortic, tricuspid and pulmonary valves, *la*, *lv*, *ra* and *rv* denote left atrium and ventricle and right atrium and ventricle, *ao*, *as*, *vs* denote aorta, systemic arterioles and systemic veins, *po*, *ap*, *vp* pulmonary arteries, pulmonary arterioles and pulmonary veins.

The circulatory system included the aorta, the systemic arteries and veins, and the pulmonary arteries and veins. Blood flow in the circulatory system was described using a lumped parameter model, which included electrical analogues for resistance (*R*), compliance (*C*) and inertia (*L*). The aortic blood pressure (*p*_*ao*_) and flow rate signals (*Q*_*ao*_) are provided below.

10 $$\frac{{dp_{ao} }}{dt} = \frac{{Q_{av} - Q_{ao} }}{{C_{ao} }}$$

11 $$\frac{{dQ_{ao} }}{dt} = \frac{{p_{ao} - p_{as} - R_{ao} Q_{ao} }}{{L_{ao} }}$$

here *Q*_*av*_ and *p*_*as*_ represent the aortic valve flow rate and systemic arteriolar pressure, respectively, and *C*_*ao*_, *R*_*ao*_ and *L*_*ao*_ representing compliance, resistance and inertance in the aorta, respectively. The electric-analogue of the cardiovascular system model is represented in Figure 1.

Ejection fraction (*EF*) in the cardiovascular system models were calculated as given below.

12 $$EF = \frac{SV}{{EDV}} \times 100$$

Here, *SV* and *EDV* are the ventricular stroke and end-diastolic volumes.

### Parameter Optimisation

**Figure 2:**
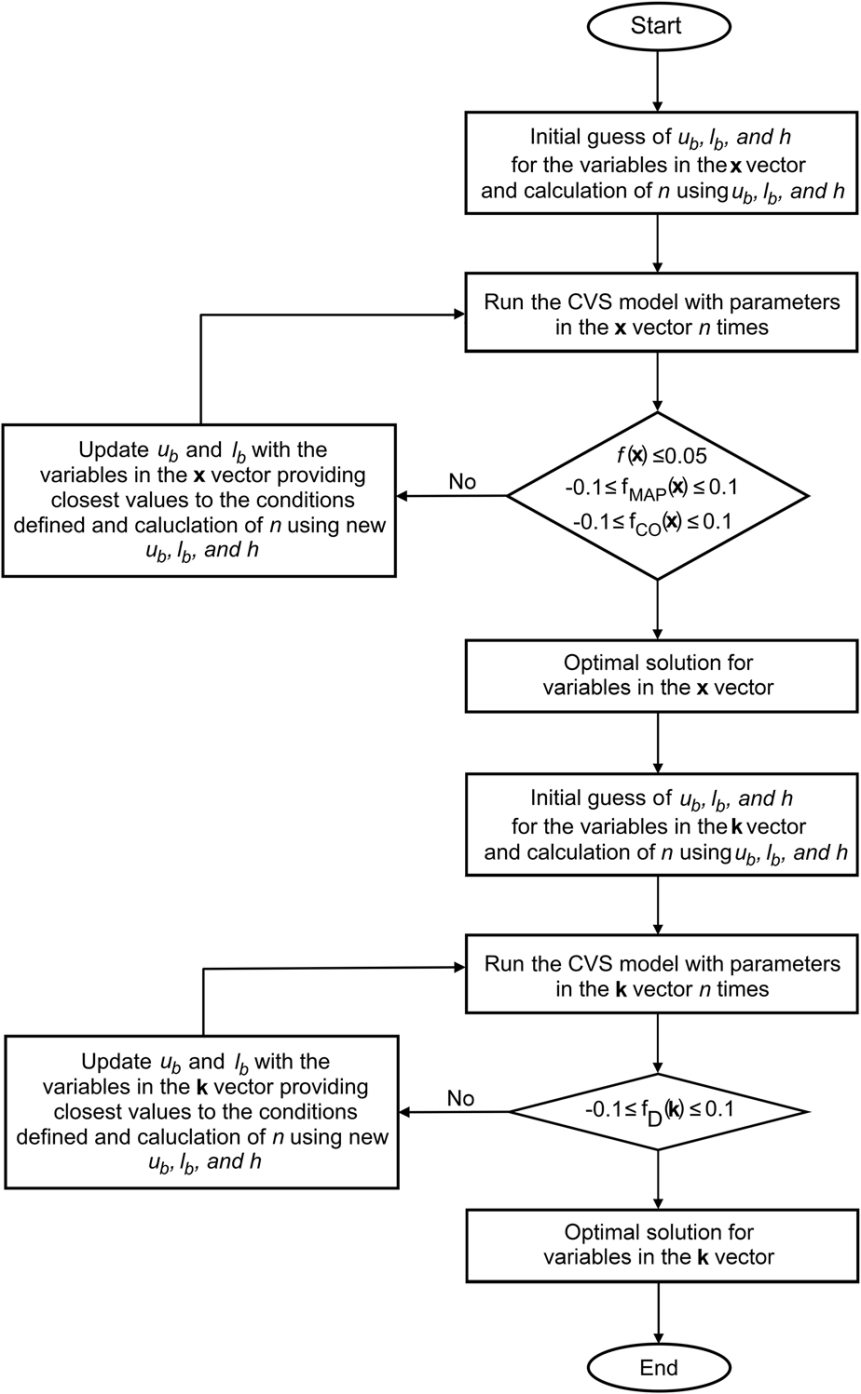
Flowchart of the optimisation algorithm.

Parameters were optimised for each patient to simulate systolic and diastolic pressures, end-systolic and end-diastolic volumes, cardiac output, and left and right ventricular diameters according to the retrieved clinical data, using a direct search method. Flowchart describing the optimisation algorithm is given in Figure 2.

The objective function (*f*) in the optimisation process aimed to minimise the difference in mean arterial pressure (*MAP*) and cardiac output (*CO*) between clinical data and the cardiovascular system model. The optimisation algorithm was designed to find a quick and effective solution for parameter estimation problem with multiple variables such as in the presented cardiovascular system model. The utilised optimisation method evaluates multiple variables in each simulation and effectively finds optimal solutions without computational cost.

The utilised electric analogue model of blood vessels simulates blood flow by solving ordinary differential equations for pressures and flow rates in each compartment. Heart chambers simulate pressure and volume which in turn affects the cardiac output the cardiovascular system. Error functions and objective function includes flow rate (*CO*) and pressure (*MAP*) terms to ensure that objective function is sensitive to the parameter set in the **x** vector. MAP in the cardiovascular system model is the time-averaged aortic pressure (*p*_*ao*_) over a cardiac cycle in each patient.

13 $$f({\mathbf{x}}) = \left| {f_{MAP} ({\mathbf{x}}) + f_{CO} ({\mathbf{x}})} \right|$$

14 $$f_{MAP} ({\mathbf{x}}) = \frac{{MAP_{pt} - MAP_{m} ({\mathbf{x}})}}{{MAP_{pt} }}$$

15 $$f_{CO} ({\mathbf{x}}) = \frac{{CO_{pt} - CO_{m} ({\mathbf{x}})}}{{CO_{pt} }}$$

here *f*_*MAP*_ and *f*_*CO*_ are the relative error functions for mean arterial pressure and cardiac output. The parameter set (**x**) included left and right ventricular end-systolic elastances (*E*_*es,lv*_, *E*_*es,rv*_), left and right ventricular zero-pressure volumes (*V*_*0,lv*_, *V*_*0,rv*_), the coefficients used in the passive pressure components of the ventricular pressures (*A*_*lv*_, *A*_*rv*_, *B*_*lv*_, *B*_*rv*_), the compliances and resistances of the aorta, systemic arteries, and veins, pulmonary artery, arterioles and veins (*R*_*ao*_, *C*_*ao*_, *R*_*as*_, *C*_*as*_, *R*_*vs*_, *C*_*vs*_, *R*_*po*_, *C*_*po*_, *R*_*ap*_, *C*_*ap*_, *R*_*vp*_, *C*_*vp*_) and the circulating blood volume (*V*_*blood*_).

16 $${\mathbf{x}} = \;\left[ {E_{es,lv} ,E_{es,rv} ,V_{0,lv} ,V_{0,rv} ,A_{lv} ,A_{rv} ,B_{lv} ,B_{rv} ,R_{ao} ,C_{ao} ,R_{as} ,C_{as} ,R_{vs} ,C_{vs} ,R_{po} ,C_{po} ,R_{ap} ,C_{ap} ,R_{vp} ,C_{vp} ,V_{blood} } \right]^{T}$$

Upper and lower bounds of the optimised parameters (**x**_**ub**_, **x**_**lb**_) were chosen around the values from the concept model published in.^3^ Step-size (*h*) in the optimisation was defined using **x**_**ub**_, **x**_**lb**_ and iteration number (*n*) in each simulation, and the parameters were updated using the initial lower bounds of the parameters and the step size, except for the coefficients *A* and *B*. These were updated starting with the upper bounds. Because arterial pressure and cardiac output decrease with the increasing A and B. Step size (*h*) and parameter updates are given in the Equations 17-19.

17 $${\mathbf{h}} = ({\mathbf{x}}_{{{\mathbf{ub}}}} - {\mathbf{x}}_{{{\mathbf{lb}}}} )/n$$

18 $${\mathbf{x}}_{{\mathbf{i}}} = {\mathbf{x}}_{{{\mathbf{lb}}}} + {\mathbf{h}}_{{\mathbf{i}}}$$

19 $${\mathbf{A}}_{{\mathbf{i}}} = {\mathbf{A}}_{{{\mathbf{ub}}}} - {\mathbf{h}}_{{\mathbf{i}}}$$

The optimisation algorithm was set to complete 10 iterations in one simulation. The upper and lower bounds of the parameters were updated according to the values of the objective and error functions. The simulations were repeated to complete 10 iterations in each simulation with updated upper and lower bounds until *f*(**x**)≤0.05, *f*_*MAP*_(**x**)≥-0.1 and *f*_*MAP*_(**x**)≤0.1, *f*_*CO*_(**x**)≥-0.1 and *f*_*CO*_(**x**)≤0.1.

The left and right ventricular diameters were estimated by optimising the coefficients in *K*_*lv*_ and *K*_*rv*_.

20 $${\mathbf{k}}\; = \;\left[ {K_{lv} ,K_{rv} } \right]^{{\text{T}}}$$

The error function for the diastolic and systolic diameters of the left and right ventricles were defined as:

21 $$f_{D} ({\mathbf{k}}) = \frac{{D_{pt} - D_{m} ({\mathbf{k}})}}{{D_{pt} }}$$

Upper and lower bounds of the **k** vector (**k**_**ub**_, **k**_**lb**_) were chosen around the values from the concept model published in.^3^ Again, the step-size (*h*) in the optimisation was defined using **k**_**ub**_, **k**_**lb**_, and the iteration number (*n*) in each simulation and the parameters were updated using the initial lower bounds of the parameters and the step size. *K*_*lv*_ and *K*_*rv*_ do not affect any other variable but ventricular diameters, depending on the ventricular volumes. Therefore, first ventricular volumes were optimized and then *K*_*lv*_ and *K*_*rv*_ were tuned according to the ventricular volumes. Initial upper and lower bound of the parameters in the **k** vector are reported in Table 2.

22 $${\mathbf{h}} = ({\mathbf{k}}_{{{\mathbf{ub}}}} - {\mathbf{k}}_{{{\mathbf{lb}}}} )/n$$

23 $${\mathbf{k}}_{{\mathbf{i}}} = {\mathbf{k}}_{{{\mathbf{lb}}}} + {\mathbf{h}}_{{\mathbf{i}}}$$

Again, the optimisation algorithm was run to complete 10 iterations in each simulation. The upper and lower bounds of the parameters were updated according to the values of the error functions. The simulations were repeated to complete 10 iterations in each simulation with updated upper and lower bounds until *f*_*D*_(**k**)>-0.1 and *f*_*D*_(**k**)≤0.1. Initial upper and lower bounds of the parameters in the **x a**nd **k** vectors are reported in Table 1.

**Table 1:** Initial upper and lower bounds of the parameters in the **x** and **k** vectors in the cardiovascular system models (*E*, V, *A* and *B* represent elastance, volume and the parameters used in the passive properties in the ventricle models, *R*, *C* and *L* represent resistance, compliance and inertance of the blood vessels, *K* is a coefficient in the ventricle models, subscripts *es*, *lv*, *rv*, *0* represent end-systole, left and right ventricles and initial value, *ao*, *as*, *vs*, *po*, *ap*, *vp* and *blood* represent aorta, systemic arteries and veins, pulmonary artery, arterioles and veins, and blood).

	Model 1		Model 2		Model 3	
	ub	lb	ub	lb	ub	lb
E_es,lv_ [mmHg/mL]	3.5	1.5	2.2	0.7	4.75	1.25
E_es,rv_ [mmHg/mL]	2	1	1.6	0.8	0.8	0.1
V_0,lv_ [mL]	10	5	25	10	10	0
V_0,rv_ [mL]	10	5	30	15	60	10
A_lv_ [mmHg]	1.3	0.9	1.2	0.8	1.4	1
A_rv_ [mmHg]	1.3	0.9	1.2	0.8	1	0.1
B_lv_ [1/mL]	0.03	0.02	0.025	0.015	0.03	0.02
B_rv_ [1/mL]	0.03	0.02	0.025	0.015	0.02	0.01
R_ao_ [mmHg s/mL]	0.058	0.038	0.17	0.12	0.51	0.01
C_ao_ [mL/mmHg]	0.273	0.173	0.273	0.173	0.3	0.1
R_as_ [mmHg s/mL]	1.1	0.5	1.4	0.9	2.5	1.0
C_as_ [mL/mmHg]	0.833	0.333	1.5	0.75	1.0	0.2
R_vs_ [mmHg s/mL]	0.058	0.038	0.058	0.038	0.11	0.01
C_vs_ [mL/mmHg]	16.35	11.35	20	12	10	5
R_po_ [mmHg s/mL]	0.012	0.007	0.012	0.007	0.11	0.01
C_po_ [mL/mmHg]	2.583	1.333	1.6	0.6	2	1
R_ap_ [mmHg s/mL]	0.158	0.138	0.158	0.138	1.5	0.1
C_ap_ [mL/mmHg]	0.103	0.053	0.153	0.103	1	0.1
R_vp_ [mmHg s/mL]	0.058	0.038	0.058	0.038	0.11	0.01
C_vp_ [mL/mmHg]	16.35	11.35	20	12	15	5
V_blood_ [mL]	750	550	700	550	650	550
K_lv_	2.0	1.0	2.0	1.0	2.0	1.0
K_rv_	4.5	1.5	3.0	1.0	2.5	0.5

Blood volume optimised in this study is the circulating blood volume. Total blood volume in children for the studied ages changes between 2000 mL and 4000 mL.^27^ However, most of the blood volume consists of unstressed blood and constitutes a blood volume reserve.^11^ Circulating blood volume is less than the total blood volume, therefore, optimised values for the circulating blood volume were less than the total blood volume in the cardiovascular system models.

Patient-specific heart rates and left and right ventricular long axis lengths (*l*_v_) were used as input values in the model. Ventricular long axis lengths were kept constant in the simulations and calculated using systolic and diastolic long axis lengths (*l*_*v,sys*_, *l*_*v,dias*_) in each patient as:

24 $$l_{v} = \frac{{l_{v,sys} + l_{v,dias} }}{2}$$

The parameters used in in the compartments describing atria, forward flow rate characteristics of the heart valves and inertance in the blood vessels were kept the same in all the models values because there were no data available to optimise them. Heart valve characteristics were assumed to be the same unless there were insufficiency. Moreover, inertial properties of blood had negligible effects on the CO and MAP. The parameter values that were not optimised for each specific case are reported in Table 2.

**Table 2:** Parameters kept the same in the cardiovascular system models (*K* is a coefficient in the atria models, *l* is longitudinal diameter of the atria, *R* and *L* are the resistance and inertance, subscripts *la*, *ra*, *mv*, *av*, *tv*, *pv*, *ao*, *as*, *ap* and *po* represent left and right atria, mitral, aortic, tricuspid and pulmonary valves, aorta, systemic arteries, pulmonary arteries and pulmonary arterioles, *f* and *b* represent forward and backward flow resistances in the heart valve models). * shows backward flow resistances in the tricuspid valve and pulmonary valves in Cardiovascular System Model simulating reverse valve flow rate in Patient 3.

Parameter	Parameter Value
K_la_	2.5
K_ra_	2.5
l_la_ [cm]	4.5
l_ra_ [cm]	4.5
R_mv,f_ [mmHg s/mL]	0.002
R_mv,b_ [mmHg s/mL]	1.00E16
R_av,f_ [mmHg s/mL]	0.002
R_av,f_ [mmHg s/mL]	1.00E16
R_tv,f_ [mmHg s/mL]	0.001
R_tv,b_ [mmHg s/mL]	1.00E16 (0.55*)
R_pv,f_ [mmHg s/mL]	0.001
R_pv,b_ [mmHg s/mL]	1.00E16 (3.5*)
L_ao_ [mmHg s^2^/mL]	1.00E-05
L_as_ [mmHg s^2^/mL]	1.00E-05
L_po_ [mmHg s^2^/mL]	1.00E-05
L_ap_ [mmHg s^2^/mL]	1.00E-05

The optimisation and simulation processes were carried out in Matlab Simulink R2017a. The set of equations was solved using the ode15s solver. The maximum step size was 1e-3 s and the relative tolerance was set to 1e-3.

## Results

Haemodynamic variables collected from the clinical database and those from the numerical models simulating each patient specific cardiac function are provided in Table 3.

**Table 3:** Input variables (*HR*, *l*_*lv*_, *l*_*rv*_) and the simulated haemodynamic variables in the patients and simulation results in the numerical models simulating patient specific cardiac function (*HR*, *MAP* and *CO* represent heart rate, mean arterial pressure and cardiac output, *p*, *V*, *SV*, *EF*, *D* and *l* represent pressure, volume, stroke volume, ejection fraction, diameter and length, subscripts *ao*, *lv*, *rv*, *sys* and *dias* represent aorta, left and right ventricles, systolic and diastolic phases respectively).

	Patient 1	Model 1	Patient 2	Model 2	Patient 3	Model 3
HR [bpm]	101	101	69	69	80	80
l_lv,sys_ [cm]	5.9	–	6.3	–	7.3	–
l_lv,dias_ [cm]	7.1	–	6.7	–	7.7	–
l_lv_ [cm]	–	6.5	–	6.5	–	7.5
l_rv,sys_ [cm]	5.1	–	6.7	–	7.1	–
l_rv,dias_ [cm]	6.5	–	7.9	–	7.3	–
l_rv_ [cm]	–	5.8	–	7.3	–	7.2
MAP [mmHg]	76.3	82.2	60.7	59.5	82.0	85.0
CO [L/min]	5.3	5.15	2.6	2.7	2.6	2.6
p_ao,sys_ [mmHg]	109	106	84	88	118	117
p_ao,dias_ [mmHg]	60	57	49	43	64	61
V_lv,sys_ [mL]	48	48	86	83	45	48
V_lv,dias_ [mL]	100	98	119	121	74	80
SV_lv_ [mL]	52	50	33	38	29	32
V_rv,sys_ [mL]	26	29	51	47	134	135
V_rv,dias_ [mL]	83	79	84	85	183	182
SV_rv_ [mL]	57	50	33	38	49	47
EF_lv_ [%]	52	51	28	31	39	40
EF_rv_ [%]	69	63	39	45	27	26
D_lv,sys_ [cm]	3.4	3.3	4.4	4.2	2.7	2.7
D_lv,dias_ [cm]	4.6	4.7	4.9	5.1	3.5	3.5
D_rv,sys_ [cm]	1.7	1.7	2.5	2.3	4.8	4.5
D_rv,dias_ [cm]	2.7	2.8	2.9	3.1	5.1	5.2

Haemodynamic values from the patients and numerical models were generally in good agreement. The largest differences were for blood pressures, in Patient 2 aortic pressure in diastole (6 mmHg); for ventricular volumes, in Patient 3 for the left ventricle in diastole (6 mL); for stroke volume, in Patient 1 for the right ventricle (7 mL). Ventricular diameters were also in good agreement, with the largest difference being 0.3 cm in diastolic right ventricular diameter in Patient 3. The upper and lower bounds of the parameters in the **x** and **k** vectors obtained from the initial optimisation and used in the second optimisation are given in Table 4.

**Table 4:** The upper and lower bounds of the parameters in the **x** and **k** vectors in the second optimisation (*E*, V, *A* and *B* represent elastance, volume and the parameters used in the passive properties in the ventricle models, *R*, *C* and *L* represent resistance, compliance and inertance of the blood vessels, *K* is a coefficient in the ventricle models, subscripts *es*, *lv*, *rv*, *0* represent end-systole, left and right ventricles and initial value, *ao*, *as*, *vs*, *po*, *ap*, *vp* and *blood* represent aorta, systemic arteries and veins, pulmonary artery, arterioles and veins, and blood).

	Model 1		Model 2		Model 3	
	ub	lb	ub	lb	ub	lb
E_es,lv_ [mmHg/mL]	2.5	2.3	1.3	1	2.65	1.95
E_es,rv_ [mmHg/mL]	1.5	1.4	1.12	0.96	0.38	0.24
V_0,lv_ [mL]	7.5	7	16	13	4	2
V_0,rv_ [mL]	7.5	7	21	18	30	20
A_lv_ [mmHg]	1.14	1.1	1.12	1.04	1.32	1.24
A_rv_ [mmHg]	1.14	1.1	1.12	1.04	0.82	0.64
B_lv_ [1/mL]	0.026	0.025	0.023	0.021	0.028	0.026
B_rv_ [1/mL]	0.026	0.025	0.023	0.021	0.018	0.016
R_ao_ [mmHg s/mL]	0.048	0.046	0.14	0.13	0.21	0.11
C_ao_ [mL/mmHg]	0.223	0.213	0.213	0.193	0.18	0.14
R_as_ [mmHg s/mL]	0.8	0.74	1.1	1	1.6	1.3
C_as_ [mL/mmHg]	0.583	0.533	1.05	0.9	0.52	0.36
R_vs_ [mmHg s/mL]	0.048	0.046	0.046	0.042	0.05	0.03
C_vs_ [mL/mmHg]	13.85	13.35	15.2	13.6	7	6
R_po_ [mmHg s/mL]	0.0095	0.009	0.009	0.008	0.05	0.03
C_po_ [mL/mmHg]	1.958	1.833	1	0.8	1.4	1.2
R_ap_ [mmHg s/mL]	0.148	0.146	0.146	0.142	0.66	0.38
C_ap_ [mL/mmHg]	0.078	0.073	0.123	0.113	0.46	0.28
R_vp_ [mmHg s/mL]	0.048	0.046	0.046	0.042	0.05	0.03
C_vp_ [mL/mmHg]	13.85	13.35	15.2	13.6	9	7
V_blood_ [mL]	650	630	610	580	590	570
K_lv_	1.40	1.20	1.50	1.30	-	-
K_rv_	1.80	1.50	1.40	1.00	1.10	0.90

The difference between the upper and lower bounds were narrowed down after the first optimisation with the initial upper and lower bounds of the parameters. The second optimisation allowed us to calculate optimal values satisfying conditions defined in the objective function and the error functions for the parameters in the **x** and **k** vectors, as given in the Table 5. The final value of the objective and error functions are provided in Table 6.

**Table 5:** Optimal values of the parameters in the **x** and **k** vector (*E*, V, *A* and *B* represent elastance, volume and the parameters used in the passive properties in the ventricle models, *R*, *C* and *L* represent resistance, compliance and inertance of the blood vessels, *K* is a coefficient in the ventricle models, subscripts *es*, *lv*, *rv*, *0* represent end-systole, left and right ventricles and initial value, *ao*, *as*, *vs*, *po*, *ap*, *vp* and *blood* represent aorta, systemic arteries and veins, pulmonary artery, arterioles and veins, and blood).

	Model 1	Model 2	Model 3
E_es,lv_ [mmHg/mL]	2.480	1.150	2.440
E_es,rv_ [mmHg/mL]	1.490	1.040	0.338
V_0,lv_ [mL]	7.450	14.500	3.400
V_0,rv_ [mL]	7.450	19.500	27.000
A_lv_ [mmHg]	1.104	1.080	1.264
A_rv_ [mmHg]	1.104	1.080	0.694
B_lv_ [1/mL]	0.025	0.022	0.027
B_rv_ [1/mL]	0.025	0.022	0.017
R_ao_ [mmHg s/mL]	0.048	0.135	0.180
C_ao_ [mL/mmHg]	0.222	0.203	0.168
R_as_ [mmHg s/mL]	0.794	1.050	1.510
C_as_ [mL/mmHg]	0.578	0.975	0.456
R_vs_ [mmHg s/mL]	0.048	0.044	0.044
C_vs_ [mL/mmHg]	13.800	14.400	6.700
R_po_ [mmHg s/mL]	0.009	0.009	0.044
C_po_ [mL/mmHg]	1.946	0.900	1.340
R_ap_ [mmHg s/mL]	0.148	0.144	0.576
C_ap_ [mL/mmHg]	0.078	0.118	0.406
R_vp_ [mmHg s/mL]	0.048	0.044	0.044
C_vp_ [mL/mmHg]	13.800	14.400	8.400
V_blood_ [mL]	638	595	584
K_lv_	1.30	1.38	1.70
K_rv_	1.68	1.20	0.90

**Table 6:** Objective function and error functions for the optimal parameter set

	Model 1	Model 2	Model 3
f	0.048	0.019	0.037
f_MAP_	− 0.076	0.019	− 0.037
f_CO_	0.028	− 0.038	0
f_Dlv,sys_	0.029	0.045	0
f_Dlv,dias_	− 0.022	− 0.041	0
f_Drv,sys_	0	0.080	0.063
f_Drv,dias_	− 0.037	− 0.069	− 0.020

**Figure 3:**
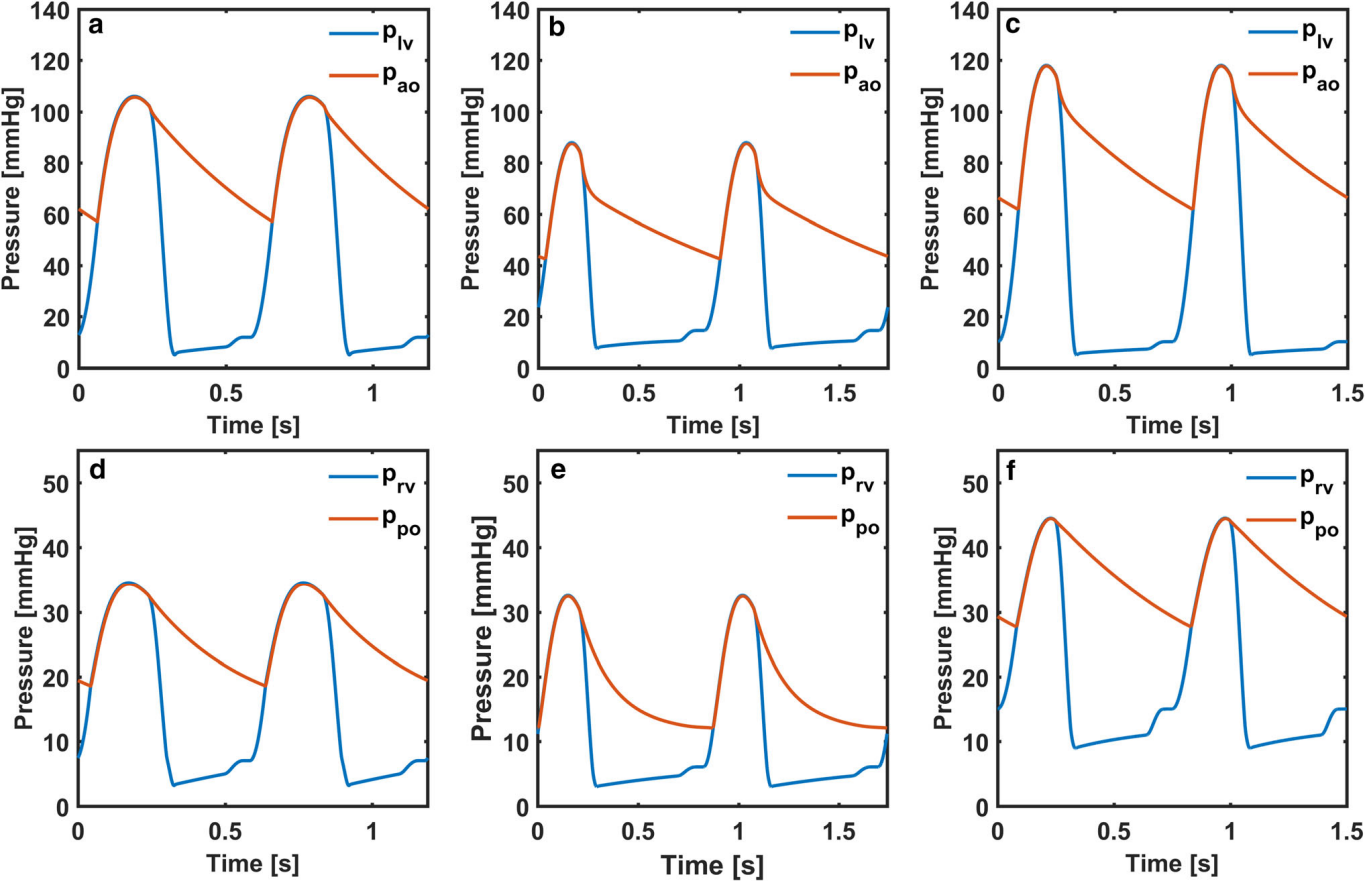
Left ventricular and aortic pressures (*p*_*lv*_, *p*_*ao*_) in the cardiovascular system models simulating cardiac function in (a) Patient 1, (b) Patient 2 and (c) Patient 3, right ventricular and pulmonary arterial pressures (*p*_*rv*_, *p*_*po*_) in the cardiovascular system models simulating cardiac function in (d) Patient 1, (e) Patient 2 and f) Patient 3.

The objective function *f* was ≤0.050 for all models in the optimisation. Moreover, the error function remained between -0.1 and 0.1. The plots of left and right ventricular pressures, and aortic and pulmonary arterial pressures from the three models are shown in Figure 3.

**Figure 4:**
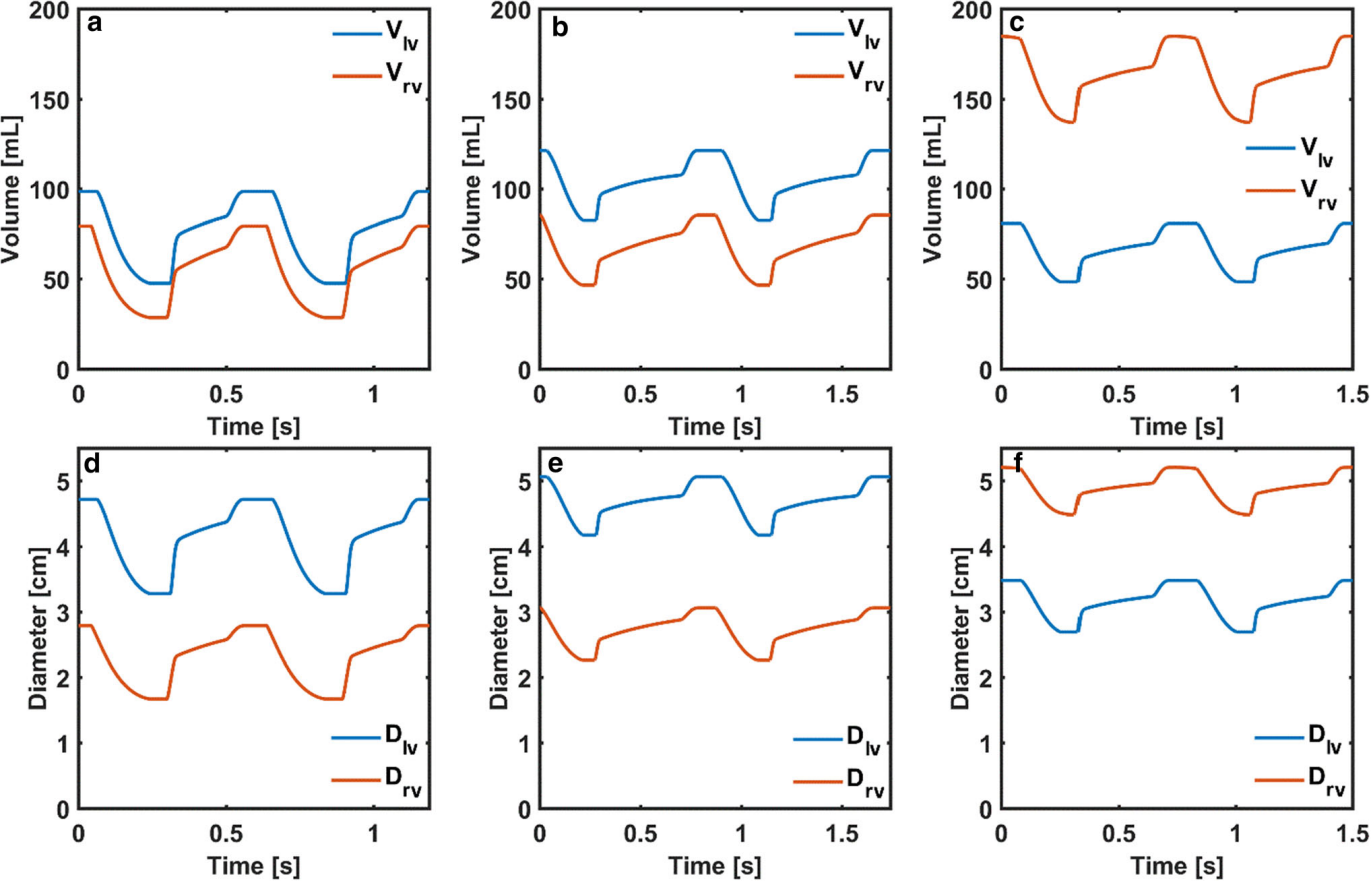
Left and right ventricular volumes (*V*_*lv*_, *V*_*rv*_) in the cardiovascular system models simulating cardiac function in a) Patient 1, b) Patient 2 and c) Patient 3, left and right ventricular basal diameters (*D*_*lv*_, *D*_*rv*_) in the cardiovascular system models simulating cardiac function in d) Patient 1, e) Patient 2 and f) Patient 3.

Systolic and diastolic aortic pressures were around 106 mmHg and 57 mmHg correlating with the patient data in Patient 1. Systolic right ventricle and pulmonary arterial pressures were around 35 mmHg whereas diastolic pulmonary arterial pressure was around 20 mmHg. Reduced systolic left ventricular and aortic pressures Patient 2 were simulated as in the patient data. Right ventricular and pulmonary arterial pressures were above 30 mmHg at systole, whilst diastolic pulmonary arterial pressure was slightly above 10 mmHg. Systolic left ventricular and aortic pressures were around 118 mmHg in Patient 3. Both systolic and diastolic pressures were remarkably high in the right ventricle showing the effects of both systolic and diastolic dysfunctions in the right ventricle. Left and right ventricular volumes and basal diameters extracted from the models are plotted over 2 cardiac cycles in Figure 4.

The left ventricular volume was larger than the right ventricular volume during the entire cardiac cycle in both Patient 1 and 2. The right ventricle was remarkably dilated in Patient 3, whereas the left ventricular volume remained below 100 mL over the cardiac cycle. Moreover, the right ventricular stroke volume was larger than the left ventricular stoke volume in Patient 3 because of the reverse flow through the tricuspid and pulmonary valves. Left ventricular basal diameter was higher than the right ventricular basal diameter in Patient 1 and 2, whilst right ventricular basal diameter was relatively high in Patient 3.

**Figure 5:**
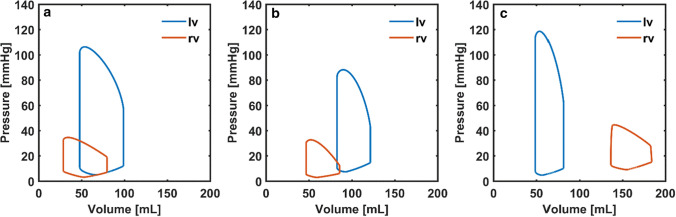
Left and right ventricular pressure-volume loops (*lv, rv*) in the cardiovascular system models simulating cardiac function in (a) Patient 1, (b) Patient 2 and (c) Patient 3.

The left and right ventricular pressure-volume loops from the models are given in Figure 5.

The left ventricular pressure-volume loop shifted to the right in Patient 1 and Patient 2 due to systolic dysfunction in the left ventricle. In Patient 3, the right ventricular pressure-volume loop shifted to the right, indicating impaired right ventricular function.

## Discussion

In this study, the parameters of a cardiovascular system model which included the heart, and the systemic and pulmonary circulations, were optimised to simulate patient specific cardiac function in 3 paediatric patients diagnosed with dilated cardiomyopathy. In children, dilated cardiomyopathy is diagnosed considering ventricular end-diastolic and end-systolic diameters indexed on body surface area, and measurements of the ventricular systolic function.^19^ Left ventricular systolic elastance (*E*_*es,lv*_) in adults is around 2.5 mmHg/mL whereas it is reported to be above 3 mmHg/mL in children between 6 and 15 ages.^13,15^ Relatively high systolic elastance values in Patient 1 and Patient 3 show the effect of a mildly affected left ventricular function; whilst for Patient 2, diagnosed with severe left ventricular cardiomyopathy, left ventricular systolic elastance (*E*_*es,lv*_) was the lowest (Table 5). A typical value right ventricular systolic elastance (*E*_*es,rv*_) is around 1 mmHg/mL in adults,^4^ whilst 1.4 mmHg/mL has been used for children between 8-12 years age.^3^ 1.040 mmHg/mL right ventricular elastance (*E*_*es,rv*_) shows impaired right ventricular systolic function in Patient 2 (Table 5). Moreover, 0.338 mmHg/mL right ventricular elastance (*E*_*es,rv*_) in Patient 3 (Table 5) shows severely impaired right ventricular systolic function, correlating with the clinical findings.

The parameters *A* and *B* were used to describe the passive ventricular pressure-volume relation. The same intervals resulting in the same optimal values were used for the left and right ventricles in the models simulating cardiac function in Patient 1 and 2, in these patients, only systolic dysfunction, which is related to active contraction behaviour in the ventricles, has been reported. Therefore, left and right ventricular passive contraction properties were assumed to be similar in these patients. Conversely, Patient 3 was diagnosed with diastolic ventricular dysfunction in both ventricles. Therefore, relatively large intervals for the coefficients *A* and *B* were selected during the optimisation. Relatively low *A* and *B* values in the right ventricle model of Patient 3 shows severely impaired diastolic function in Patient 3, along with the systolic dysfunction.

Systemic vascular resistance increases in heart failure as a response to neurohumoral pathways to maintain perfusion level.^17^ Increased systemic arteriolar resistance (*R*_*as*_) can be seen in Patient 2 and 3 as the cardiac output and mean arterial pressure in these patient were relatively low. Upper and lower bounds of the other parameters were selected considering the concept model and haemodynamic variables such as ventricular volumes and arterial pressures reported in the patient clinical data. For instance, the interval between the upper and lower bounds in the ventricular zero-pressure volumes (*V*_*0*_) were selected higher for the relatively high ventricular volumes clinically reported. Moreover, the right ventricular zero-pressure volumes (*V*_*rv,0*_) were higher in the concept model reported in.^3^ Therefore, ventricular zero-pressure volumes (*V*_*0*_) were the same for left and right ventricles in the cardiovascular system model simulating Patient 1 where right ventricular zero-pressure volumes (*V*_*rv,0*_) were higher as in the cardiovascular system model simulating Patients 2 and 3.

The **x** vector contained 21 parameters and the **k** vector contained 2 parameters. The optimal solution depends on the selected upper and lower bounds (*ub*, *lb*), and on the step-size (*h*). It may be possible to obtain the global solution or local minima which provide better solutions by changing the initial upper and lower bounds, step size or interval for the error functions. However, it should be noted that the aim of the study was to simulate clinical conditions within the specified limits for the error functions (*f*_*MAP*_ and *f*_*CO*_) and show that it could be possible to simulate clinical values in the patients using lumped parameter models. Also, relatively small step-sizes may allow to find an optimal solution in one run, whilst larger step-sizes updates the upper and lower bounds of the parameters and require running the optimisation algorithm more than once. Therefore, an initial simulation would be required for relatively large upper and lower bound intervals to narrow down the intervals for lower and upper bounds of the parameters. A numerical model utilising relatively large intervals for upper and lower bounds would take more time to find an optimal solution. The used optimisation method allowed us to optimise a parameter set containing several parameters without additional computational cost, as it utilised the parameter values simultaneously to find the optimal solution. Therefore, it is also suitable for low configuration computers.

Ventricular passive pressure component uses left ventricular volume as its independent variable. Although the ventricular pressures may not be zero at the specified zero-pressure volumes (*V*_*0*_) values, the utilised model simulate left ventricular pressures within the physiological limits. Utilising also *V*_*0*_ in the ventricular passive pressure component will allow ventricular pressures to become zero at the specified *V*_*0*_ values.^21^

Patient-specific lumped parameter models simulating cardiac function in children can be used to evaluate several different scenarios. In this study, dilated cardiomyopathy was considered, but the framework presented could be easily adapted to simulate for example restrictive or hypertrophic cardiomyopathy.^32^ Another application would be simulations of therapies, such as ventricular assist device support that are becoming a viable treatment option for end-stage heart failure paediatric patients,^30^ albeit often causing complications which may require additional intervention.^8^ Computational techniques have been utilised widely to evaluate clinical scenarios in children.^23^ In those cases, where flow and pressure data cannot be retrieved from conventional imaging modalities, patient specific lumped parameter models, as presented in this study, can be used to simulate patient specific boundary conditions for finite element models.

## Conclusions

This study shows the potential of patient specific lumped parameter models to simulate clinical cases of dilated cardiomyopathy in children. Modelling patient specific cardiac function and blood flow in paediatric patients allows assessment of a variety of conditions for better understanding of the complex hemodynamic and cardiovascular function in these cases.

## Abbreviations

p: Pressure
Q: Flow rate
V: Volume
R: Resistance
L: Inertance
C: Compliance
E: Elastance
A: Coefficient
B: Coefficient
K: Coefficient
D: Diameter
r: Radius
l: Length
MV: Mitral valve
AV: Aortic valve
TV: Tricuspid valve
PV: Pulmonary valve
EF: Ejection fraction
SV: Stroke volume
EDV: End-diastolic volume
CO: Cardiac output
MAP: Mean arterial pressure
f: Function
h: Step
n: Iteration number

## Subscripts

la: Left atrium
lv: Left ventricle
ra: Right atrium
rv: Right ventricle
ao: Aorta
as: Systemic arterioles
vs: Systemic veins
po: Pulmonary arteries
ap: Pulmonary arterioles
vp: Pulmonary veins
mv: Mitral valve
av: Aortic valve
tv: Tricuspid valve
pv: Pulmonary valve
b: Backward
f: Forward
es: End-diastolic
sys: Systolic
dias: Diastolic
v: Ventricle
act: Activation
0: Zero (pressure)
a: Active
p: Passive
ub: Upper bound
lb: Lower bound
m: Model
pt: Patient
blood: Blood
